# Engineering a Ca^++^-Sensitive (Bio)Sensor from the Pore-Module of a Potassium Channel

**DOI:** 10.3390/s150304913

**Published:** 2015-02-27

**Authors:** Mattia Lorenzo DiFrancesco, Sabrina Gazzarrini, Cristina Arrigoni, Giulia Romani, Gerhard Thiel, Anna Moroni

**Affiliations:** 1 Department of Biosciences, University of Milan, Via Celoria 26, Milano 20133, Italy; E-Mails: mattia.difrancesco@hotmail.it (M.L.D.); sabrina.gazzarrini@unimi.it (S.G.); cristina.arrigoni@ucsf.edu (C.A.); 2 Institute of Biophysics-Milan section, National Research Council, Via Celoria 26, Milano 20133, Italy; E-Mail: giulia.romani@unimi.it; 3 Department of Biology, Technische Universität Darmstadt, Schnittspahnstrasse 3, Darmstadt 64287, Germany; E-Mail: thiel@bio.tu-darmstadt.de

**Keywords:** biosensor, Kcv, calcium, calmodulin, synthetic ion channel

## Abstract

Signals recorded at the cell membrane are meaningful indicators of the physiological *vs.* pathological state of a cell and will become useful diagnostic elements in nanomedicine. In this project we present a coherent strategy for the design and fabrication of a bio-nano-sensor that monitors changes in intracellular cell calcium concentration and allows an easy read out by converting the calcium signal into an electrical current in the range of microampere that can be easily measured by conventional cell electrophysiology apparatus.

## 1. Introduction

Ion channels are pore-forming proteins found on the membrane of cells. They allow the controlled and selective flow of ions across the otherwise impermeable lipid bilayer. Ions flowing through the channel pores generate an electrical current that establishes a membrane potential that controls key cellular functions such as action potential firing and hormone release. Indeed, they behave as molecular switches, being gated (open and closed) by several factors, such as ligands, voltage and light which they perceive by means of peripheral sensor modules connected to the pore. They are able to sense ligands or physical factors with a very high selectivity and sensitivity (in the nanomolar range) and convert this into measurable electrical signals. Moreover, they produce a huge intrinsic-gain factor of about 1 million, since one molecule of ligand opens a channel that can pass one million ions (10^−6^ ions/s, 1 pA) [1]. Ion channels are promising building elements in lab-on-a-chip technologies or in diagnostic devices [2,3,4], despite the fact that their application in technical devices is still hampered by a number of factors. One of the main problems is that many channel proteins are not sufficiently robust for these applications, and they easily lose their functionality when extracted from the native membranes. Another key point is that the sensitivity of natural channels is limited to those ligands that are relevant to the physiology of cells. A possible strategy to overcome these limitations is therefore to engineer more durable channels with new sensory properties. For what concerns the first point, robust ion channels can be found in viruses. In particular plant viruses belonging to the family of *Phycodnaviridae* are a source of channel proteins known to be extremely stable. From our experience we know that, once extracted from their natural membranes, these channels retain activity for years. As for the second point, we have already shown that this class of channels are ideal building blocks in the construction of synthetic channels [5]. Such an engineering approach, which could widen the scope of sensing, is fostered by the fact that channels are modular in nature. They are composed of a central pore domain, the ion conducting element, connected to peripheral sensor domains. Perception of the stimulus occurring at the sensor domain, causes a conformational change in the sensor domain itself that is mechanically transmitted to the pore domain and here in particular to the gates. In this way conformational changes in the sensor induce gating of the pore, modulating the ion transport activity of the channel.

Here we examine whether it is possible to convert a small and robust K^+^ channel, which has no inherent Ca^++^ sensitivity, into a Ca^++^ sensitive channel. This proof of concept study should reveal whether a naïve coupling of calmodulin, (CaM, an abbreviation for CALcium-MODULated protein) a calcium-binding messenger protein expressed in all eukaryotic cells, and a K^+^ channel pore is already sufficient for lending Ca^++^ sensitivity to a synthetic channel.

**Figure 1:**
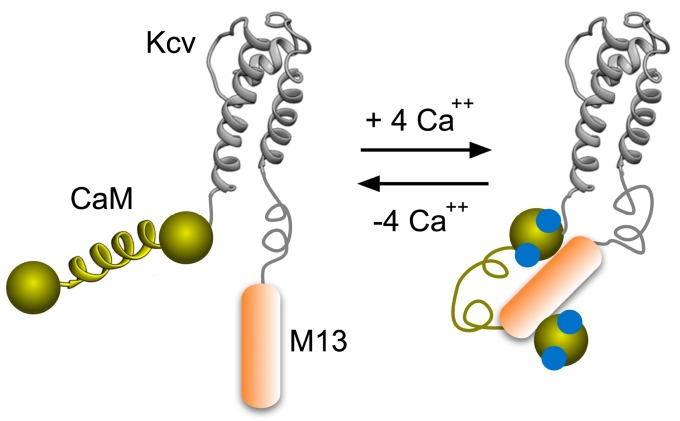
Cartoon representation of the expected mode of operation of the biosensor. A single Kcv subunit fused to the calcium-modulated protein CaM and its interacting peptide M13 is shown in the Ca^++^-unbound (**left**) and in the bound (**right**) state. When four Ca^++^ ions bind to the four CaM binding sites, the sensor protein undergoes a conformational change and enfolds the M13 peptide. The molecular rearrangement of CaM and M13 affects the Kcv pore properties leading to a change in measurable current.

The calcium sensor CaM contains four EF-hand motifs, each of which binds a Ca^++^ ion. Upon calcium binding, CaM undergoes a major conformational change and exposes a hydrophobic surface that recognises and tightly binds to the amphipathic alfa helices of several target proteins. M13 is a helical peptide chain from such a target protein, namely the myosin light chain kinase. The CaM/M13 interaction has been used before in other kind of engineered protein biosensors [6,7]. As the ion conducting pore, we use the small ion channel protein Kcv from the *Chlorella* Virus PBCV-1 [8,9,10]. The Kcv monomer, which is made by 94 amino acids, forms in eukaryotic cells a functional homotetrameric potassium (K^+^) channel [11]. The Kcv channel has the typical properties of mammalian potassium channels. These include cation selectivity, gating and sensitivity to specific blockers [12,13,14,15]. Moreover, Kcv does not lose activity when inserted in artificial membranes [16]. Kcv is the simplest possible molecular K^+^ channels and it does not include peripheral sensor domains. Conformational changes of sensing domains artificially connected to them, could in principle induce a structural rearrangement of Kcv, and possibly modulate one or more of its gating mechanisms. Indeed, previous work has already shown that the fusion of Kcv with the voltage-sensing domain of *Ciona intestinalis* (Ci-VSD) was successfully converting the voltage-insensitive Kcv channel into a voltage-gated outwardly rectifier [5]. The present data show that a similar approach was also successful for engineering a sensor for intracellular calcium by linking Kcv to the calcium-binding protein CaM and its interacting peptide (M13) (Figure 1). The positive outcome of this proof of principle study paves the way for a construction of channels with sensor properties, which are not yet present in natural channels. 

## 2. Experimental Section 

### 2.1. Chimeric Constructs

All chimeric constructs and mutants were prepared by overlapping PCR and inserted into BamHI and XhoI restriction sites of pSGEM vector (a modified version of pGEM-HE). Calmodulin and M13 were amplified from the cameleon construct D3cpv [7]. *In vitro* transcription was performed on linearized plasmids using T7 RNA polymerase (Promega, Madison, WI, USA). cRNAs were injected (50 ng per oocyte) into *Xenopus laevis* oocytes, as reported previously [11]. Electrophysiological measurements were made 3 to 4 days after injection. The CaM construct mutated in the EF hands was kindly provided by Daniel Minor (U. of California San Francisco). 

### 2.2. Electrophysiological Measurements

Two-electrode voltage-clamp (TEVC) experiments were performed as described previously [11] using GeneClamp 500 amplifier (Axon Instruments, Sunnyvale, CA, USA). Data were filtered at 1 kHz and stored at 5 kHz with pCLAMP8 software (Axon Instruments). Electrodes were filled with 3 M KCl and had a resistance of 0.2 to 0.8 MΩ in 50 mM KCl. The oocytes were perfused at room temperature, 2 mL/min with a (bath) solution containing 50 mM KCl, 1.8 mM CaCl_2_, 1 mM MgCl_2_, and 5 mM HEPES, adjusted to pH 7.4 with KOH. Mannitol was used to adjust the osmolarity of the solution to 215 mosmol/L. Voltage-Clamp protocols consisted of 20 mV steps from −200 mV to +100 mV. The holding voltage was −20 mV.

The calcium-sensitivity of the different constructs was measured at a constant voltage of −80 mV. An increase in the cytosolic calcium concentration was induced by addition of calcium ionophore ionomycin (IM) 1 µM to the external medium [17].

For single-channel recordings, the vitelline membrane of the oocytes was removed mechanically before the experiment after keeping the oocyte for 5 min in a hyperosmotic solution (ND96 solution plus 100 mM NaCl). Patch pipettes were pulled from thin-walled borosilicate glass capillaries, coated with Sylgard (Corning, Midland, MI, USA), and fire-polished to a final resistance of 2 to 5 MΩ. Bath and pipette solution contained 100 mM KCl, 1.8 mM CaCl_2_, 1 mM MgCl_2_, and 10 mM HEPES, adjusted to pH 7.4 with KOH. Currents were recorded in inside-out configuration with a Dagan 3900 amplifier (Minneapolis, MN, USA) at a sampling rate of 10 kHz, and low-pass filtered at 2 kHz.

## 3. Results and Discussion

### 3.1. Calcium Sensitivity of Kcv-Based Chimeric Constructs

**Figure 2:**
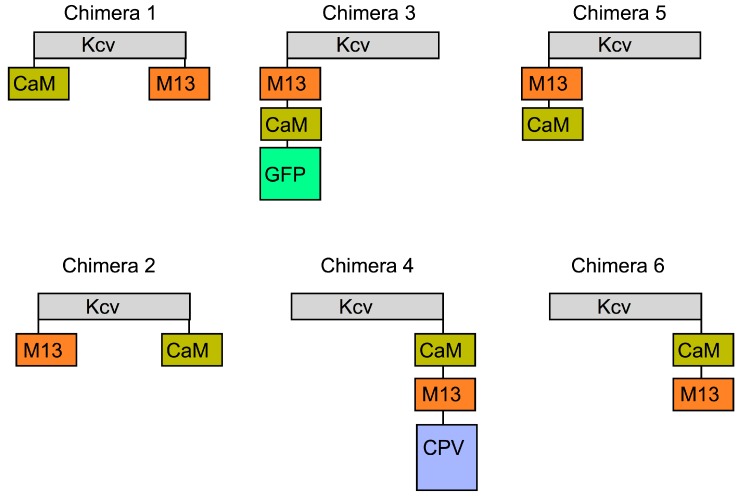
Schematic representation of the chimerae. Chimerae 1 to 6 were constructed by fusing the Kcv monomer (grey box) with CaM and its interacting peptide M13 in different positions. Chimera 3 and 4 contained also the Cyan Fluorescent Protein (CFP) and the Circularly Permuted Venus peptide (CPV) respectively.

Six chimerae, labelled 1 to 6, were designed by fusing the Kcv subunit with the calcium-binding protein calmodulin (CaM) and its interacting peptide M13 in different positions (Figure 2). The cRNA of all constructs were *in vitro* synthetized and injected in *Xenopus* oocytes. Water was injected in oocytes for control. After three days the macroscopic (macro) currents were measured from the intact oocytes by means of the two- electrode voltage clamp (TEVC) technique. Currents in the range of µA could be measured from oocytes injected with Chimera 2, 4 and 6, while the other three constructs were apparently not functional and were not analysed further.

As illustrated in Figure 2, Chimera 2 was constructed by fusing the CaM domain at the C-terminus of the second transmembrane domain (TM2), and the M13 peptide at the N-terminus of the first transmembrane domain (TM1); Chimera 6 contains both the CaM and the M13 peptide on the C-terminus of the Kcv subunit; finally Chimera 4 was constructed as Chimera 6 but with a C terminus CPV domain, a circularly permutated variant of the fluorescent protein GFP [18].

**Figure 3:**
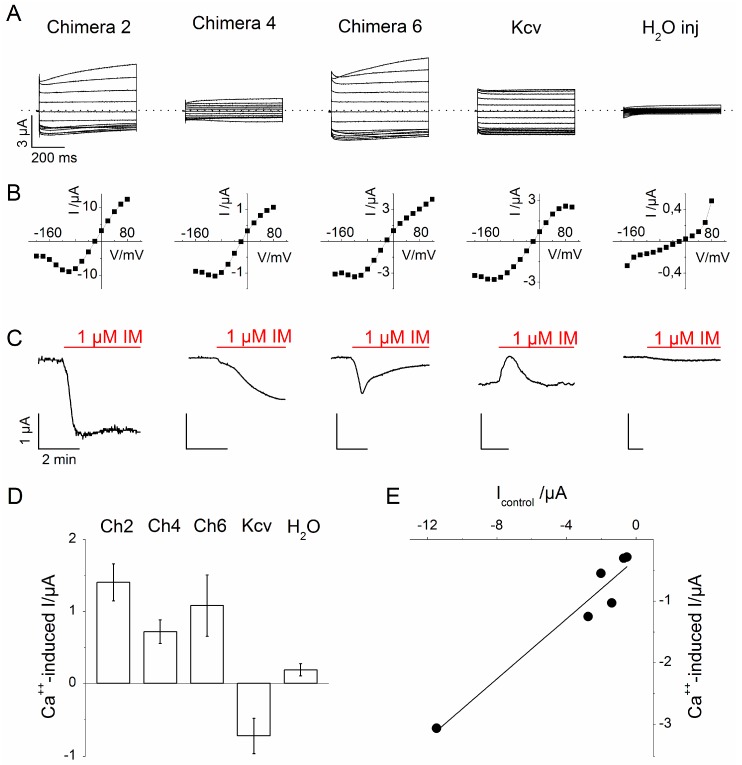
Summary of the main characteristics of the tested chimeras in comparison with Kcv wt and water injected oocytes. (**A**, upper row) Whole-cell macro currents recorded from the different constructs by two-electrode voltage clamp. Voltage protocol: from −200 to +100 mV, in 20 mV steps. (**A**, middle row) corresponding I/V curves of the above recordings. (**A**, lower row) Representative current traces recorded at −80 mV before and after (red line) the addition of IM (1 µM) to the external medium; (**B**) Bar graph showing the average current variation induced by IM in the different constructs (Chimera 2, *n* = 7; Chimera 4, *n* = 4; Chimera 6, *n*= 6; Kcv wt, *n* = 5; water injected, *n* = 6); (**C**) IM-induced (peak) current increase, plotted as a function of the initial current. Data are shown only for Chimera 6. Measurements performed at −80 mV. (**D**) Bar graph illustrates the average current variation induced by the increase in cytosolic calcium concentration for the different constructs (N: Ch2 = 7, Ch4 = 4, Ch6 = 6, Kcv = 5, H_2_O = 6); (**E**) IM-induced (peak) current increase, plotted as a function of the initial current. Data are shown only for Chimera 6. Measurement were performed at -80 mV.

Figure 3 summarizes the properties of the three chimerae, 2, 4 and 6, that produced measurable currents in *Xenopus* oocytes. Figure 3A, top row, shows that the currents recorded from the three functional constructs look similar to those of Kcv, but clearly different from the endogenous currents recorded from the water-injected oocyte. Figure 3A, middle row, shows the corresponding current-voltage (I/V) relationships, obtained from the above recordings. Not surprisingly, the chimerae show the typical Kcv feature, a negative slope conductance at voltages more negative than −100 mV. The bottom row of Figure 3A shows the response of the three constructs to changes in the intracellular calcium concentration. In this case, the current was recorded at the constant voltage of −80 mV and the sensitivity to calcium was tested by measuring the changes induced in the current by the addition of Ionomycin (IM) to the external solution. IM is an antibiotic molecule produced by the bacterium *Streptomyces conglobatus*. It acts as a Ca^++^ ionophore, allowing the transport of Ca^++^ ions across the membranes, and it is used to raise experimentally the intracellular level of Ca^++^ [17]. Typically added to the external solution of cells at the concentration of 1 μM, it induces a raise in the internal Ca^++^ that equilibrates with the external Ca^++^ concentration (mM in our experimental conditions).

As shown in 3A, bottom row, all three chimeric constructs responded to the addition of IM with an increase in current, which was nevertheless variable in amplitude and kinetics. In contrast, in oocytes expressing Kcv alone the same treatment with IM caused a transient reduction in current. This transient effect of high internal Ca^++^ on Kcv channels is of unknown origin and since it is opposite to that of the chimerae we did not studied it further. Finally, the endogenous currents from control, water-injected oocyte, did not respond to IM. 

More in details, Chimera 2 showed the highest increase in inward current upon IM addition, in average of 1.4 ± 0.26 µA (*n* = 7). The current increase was rapid (t_1/2_ = 21.6 ± 1.2 sec) and was reversed by the removal of the calcium ionophore from the bath solution (not shown). Chimera 4 displayed a smaller response to IM with a mean value of 0.72 ± 0.2 µA, (N = 4). This increase in current was also slower than that of Chimera 2 (t_1/2_ = 3.01 ± 0.2 min). In the case of Chimera 6, the calcium-induced increase in inward current was 0.8 ± 0.43 µA (*n* = 6). Also this current increase occurred with a fast kinetics but, differently from that of Chimera 2, was transient: the current returned slowly back to the baseline value in the presence of IM.

The IM-induced variations in current amplitudes for the different Chimeras are summarized in Figure 3B. In the graph of Figure 3C the calcium-induced increase in current, recorded from Chimera 6, was plotted as a function of the value of the initial current recorded at the same voltage of −80 mV. The linear relation between the plotted parameters confirms that the IM-induced current increase was specifically due to the expressed construct and not to aspecific effect of IM on endogenous oocyte current (as already shown by the water-injected oocyte, panel A, lower row).

### 3.2. Analysis of the Chimera 2 and Control Constructs

**Figure 4:**
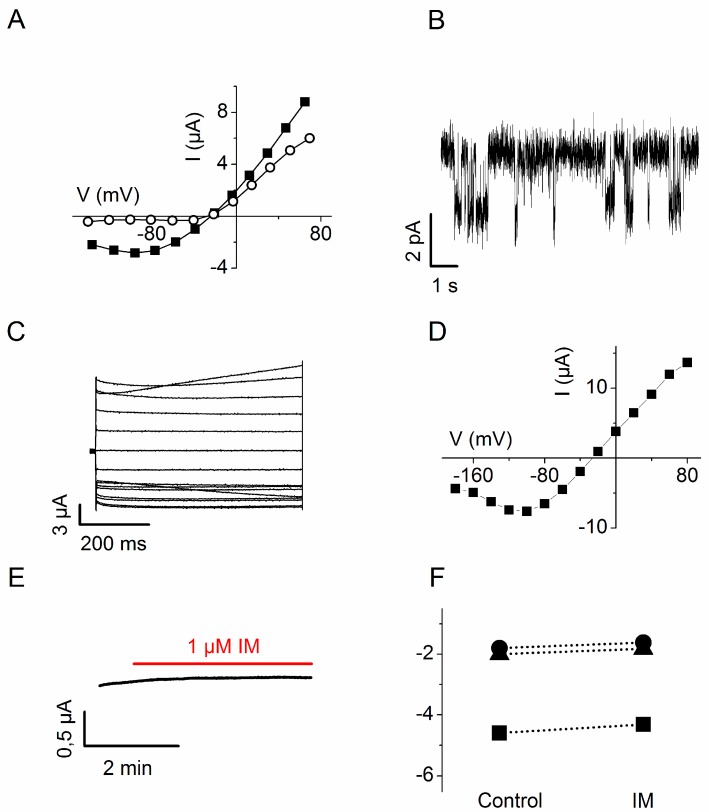
Characterization of Chimera 2 wt and properties of Chimera 2-CaM-mutated. (**A**) I/V curve of Steady State whole-cell currents recorded from the Chimera 2 in the presence of IM, before (solid square) and after (open circle) the addition of 1 mM barium in the external medium; (**B**) Single channel recording of Chimera 2 at −60 mV; (**C**) Properties of Chimera 2-Cam-mutated: whole-cell macro currents recorded with TE-Voltage Clamp; (**D**) I/V curve of steady state macro currents of Chimera 2-Cam-mutated recorded in C; (**E**) Whole-cell current variation measured at −80 mV after the addition of IM in the external medium; (**F**) Same experiment as in E, from three different oocytes.

Next, Chimera 2 was chosen for a more detailed analysis that included a characterization of the pore properties of Chimera 2 and the proof that the effect of IM is truly due to Ca^++^ binding to the chimeric channel. To confirm that the pore properties of the Chimera are unchanged with respect to the original channel Kcv, we have tested the effect of barium, a well-known inhibitor of the Kcv channel, on the IM-induced current. The data in Figure 4A show the I/V relation of a representative experiment in which the current was recorded in the constant presence of IM, with and without 1 mM BaCl_2_ in the medium. These data show that Ba^++^ is an effective blocker of the inward Kcv current, while it has less effect on the outward current (Figure 4C). Since Ba^++^ has the same effect on Chimera 2 currents [11], we can conclude that the IM-triggered currents are indeed conducted by the Kcv pore of Chimera 2 whose properties are unchanged from that of the parental channel Kcv.

Further characterization of Chimera 2 included recordings of single channel currents from oocytes expressing the channel. Figure 4B shows channel fluctuations recorded at −60 mV. From the amplitude of the current we can estimate a single channel conductance in the range of 100 pS, a value similar to the unitary conductance of Kcv [15,16].

In order to confirm that IM-triggered increase in current was a direct consequence of calcium binding to the CaM module in the chimera, we replaced the wt CaM moiety in Chimera 2 by a Ca^++^ insensitive mutant. In this construct, glutamic acids at positions 32, 68, 105 and 141 in the four calcium binding sites of the EF-hand of CaM were mutated to alanine to corrupt Ca^++^ binding [19,20]. Expression of this construct (Chimera 2-CaM-mutated) in oocytes elicited, under voltage clamp conditions, currents with kinetics (Figure 4C) and I/V relationship (4D) similar to those previously shown for the wt construct (see Figure 3A). As expected, when cytosolic calcium was increased by addition of IM, the whole-cell current recorded at −80 mV failed to show an increase (Figure 4E,F). The results of these experiments show that a Kcv/CaM chimera becomes insensitive to excursions in the cytoplasmic Ca^++^ concentration when the Ca^++^ binding property of CaM is impaired.

### 3.3. Molecular Improvement of Chimera 2

In order to improve the control of the CaM module over the Kcv pore, we have introduced rational changes on the sequence of Chimera 2 gene, guided by our previous knowledge of Kcv and of other Kcv-based chimeric channels.

**Figure 5:**
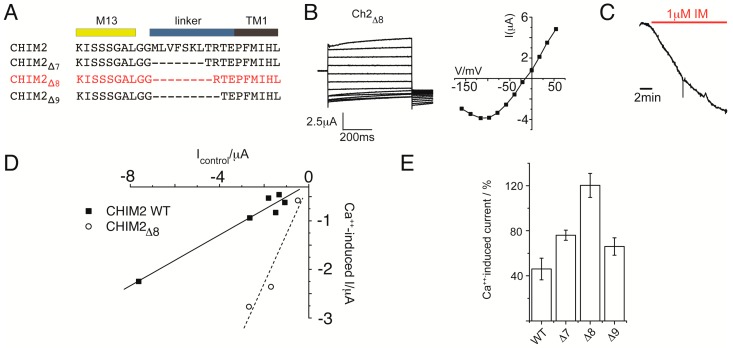
Molecular improvement of Chimera 2- (**A**) sequence alignment of the linker region in Chimera 2 (Chim2) and in the deletion mutants (Chim2_Δ__7_ Chim2_Δ8_ Chim2_Δ9_, lacking 7, 8 and 9 residues in the linker region (also known as Slide helix) connecting M13 sequence at the first transmembrane domain of Kcv (TM1); (**B**) left, exemplary current traces recorded from Chim2_Δ__8_ by two-electrode voltage clamp applying the same voltage protocol of Figure 3. The corresponding I/V relationship is shown in the same panel (right); (**C**) IM-induced current increase recorded in Chim2_Δ__8_ measured under constant voltage clamp at −80 mV; (**D**) Ca^++^-induced current increase measured in Chim2 (black square) and Chim2_Δ__8_ (open circle) plotted as a function of the initial current value (recordings obtained in all cases at −80 mV); (**E**) comparison of Ca^++^-induced current increase (%) in the three deletion chimera (Δ7, Δ8 and Δ9) and Chim2 (wt).

Previous characterization of Kcv showed that the fourteen aminoacid sequence at the N-terminal of the channel (the so-called slide-helix) plays an important role in the control of channel gating. In particular, deletion of the first seven aminoacids of the N-terminus is well tolerated by the channel, but if the deletion is extended to the threonine in position 8, Kcv wt is not functional anymore. Deletion in the slide helix of Kcv have been also tested on a different Kcv chimera, where the channel was fused to the voltage sensor domain of the Ci-VSP, the voltage sensitive phosphatase isolated from *Ciona intestinalis* [5]. In that case, partial deletions of the N-terminus slide helix improved the coupling between the voltage sensor domain and the Kcv-pore. Inspired by these observations, we designed and tested constructs of Chimera 2 with deletions of the first seven, eight and nine aminoacids of the N-terminus, Chim2, Chim2_Δ7_, Chim2_Δ8_, Chim2_Δ9_, respectively (Figure 5A). Overexpression of the three constructs in oocytes resulted in functional channels, and a representative current trace and voltage-current relationship are shown for the construct with the eight aminoacids deletion (Figure 5B): kinetics and voltage dependence resembled those of the original Chimera 2. However the Ca^++^-induced current, triggered by the perfusion of IM in the bath solution (Figure 5C), is larger than the one recorded from Chim2. In fact, the graph in Figure 5D, representing the amplitude of the Ca^++^-induced current over the basal current, showed a steeper linear correlation of the data points recorded from Chim2_Δ8_ compared to the full-length construct. Moreover of the three tested constructs, the deletion of the first eight aminoacids conferred the best improvement to the Ca^++^ response of the channel, as the current increase percentage showed in the histogram of Figure 5E (Ca^++^-induced current increase: Chim2 (46% ± 9%), Chim2_Δ__7_ (76% ± 4%), Chim2_Δ__8_ (120% ± 10%), Chim2_Δ__9_ (76% ± 7%)).

## 4. Conclusions

In previous work it was shown that the CaM/M13 interaction can be used to engineer a protein-based and genetically encoded calcium sensor, the so-called Cameleon [6,7]. Selective and high affinity binding of Ca^++^ triggers in this system an interaction between the calcium binding protein and M13. The present study demonstrates that the same Ca^++^-triggered conformational change induced by the CaM/M13 interaction can be used to gate a simple K^+^ channel pore. We show here, by prove of principle experiments, that it is possible to design ion channel-based sensors on demand. The robust small pore of the Kcv channel, which is well understood with respect to its structure/function correlates [11,12,13,14,15,16], is the ideal building block for engineering such sensors. We have shown that the distinct fusion of a sensory domain, which undergoes a conformational change upon binding of a ligand, is able to affect pore gating. More importantly, the properties of the prototype Chimera 2 can be improved by means of protein engineering. In our experience the linker sequence that connects two protein modules plays a crucial role in regulating their functional connection. As expected, reducing the length of the linker improved by a factor of two the control exerted by CaM on the pore of Chimera 2 (Figure 5E).

In future work the simple engineering strategy illustrated here, could be used to create other kind of ion channel-based biosensors which can be engineered to respond to relevant physical stimuli such as light and/or magnetic field. These kind of channel-based biosensors are highly desirable tools because they combine a high affinity biological recognition mechanism with physical transduction techniques that greatly amplifies the signal. These kinds of biosensors will be used for screening of drugs or chemicals or even in the environmental screening for pollutants. Besides, there is a strong biomedical interest in these biosensors that concerns their usage as miniaturized devices for the screening of molecule of biomedical interest [21] and even more interesting, the possibility to use ion channels to control neuronal activity by using non-invasive techniques, such as light [22].
